# Molecular Characterization of Antibiotic Resistance and Genetic Diversity of *Klebsiella pneumoniae* Strains

**DOI:** 10.1155/2022/2156726

**Published:** 2022-06-21

**Authors:** Bahareh Arabzadeh, Zeynab Ahmadi, Reza Ranjbar

**Affiliations:** Molecular Biology Research Center, Systems Biology and Poisonings Institute, Baqiyatallah University of Medical Sciences, Tehran, Iran

## Abstract

The aims of this study were the molecular characterization of antibiotic resistance and genotyping of *Klebsiella pneumoniae* strains isolated from clinical cases in Tehran, Iran. A total of 100 different types of clinical human samples were collected from a major teaching hospital in Tehran, Iran. Bacterial isolates were identified using standard microbiological tests. Antimicrobial susceptibility testing was done according to the latest CLSI guidelines. PCR was used to amplify the *gyrA* gene in quinolone-resistant isolates and sequencing was performed for the detection of probable mutations between the isolates. The occurrence of plasmid-mediated quinolone resistance genes (*qnrA*, *qnrB,* and *qnrS*) was also investigated by PCR. Finally, genotyping of the strains was performed by PFGE in a standard condition. The susceptibility pattern revealed a high and low level of resistance against meropenem (20%) and trimethoprim (37%), respectively. PCR and sequencing detected mutation in the *gyrA* gene in 51% of quinolone-resistant *K. pneumoniae*. According to the susceptibility report, among nalidixic acid-resistant strains, 60.5%, 50%, and 42.9% of isolates contained *qnrA*, *qnrB,* and *qnrS*, respectively. Among ciprofloxacin-resistant strains, *qnrA* was the most frequent PMQR gene. The PFGE differentiated the strains into 31 different genetic clusters so that the highest number (7/66) was in category *A*. Our results indicated that the frequency of resistance to various antibiotics particularly trimethoprim, nalidixic acid, and cefoxitin are increasing. The presence of *qnr* (*S* and *A*) genes and point mutation of the *gyrA* gene were likely to be responsible for the resistance toward nalidixic acid and ciprofloxacin in our strains. Also, the results obtained from genotyping indicated that the *K. pneumoniae* strains isolated in this study belonged to the diverse clones.

## 1. Introduction

*Klebsiella pneumoniae* is one of the most common pathogens in hospitals with high mortality rates and causes a variety of infections including pneumonia, urinary tract infections, septicemia, diarrhea, liver abscess, endophthalmitis, meningitis, and bacteremia [1, 2].

The rapid emergence of drug resistance to *K. pneumoniae* isolates is one of the serious problems of antibiotic treatment and causes a lot of worries in the world [3]. The availability of new information on antibiotic resistance patterns in bacterial agents can help us treat *K. pneumoniae* infections more efficiently. The *qnr* genes are one of the plasmid-mediated quinolone resistance agents, which cause very rapid resistance to Enterobacteriaceae due to their placement on different integrons [4–6]. Quinolones, such as nalidixic acid and fluoroquinolones, inhibit DNA replication through binding to topoisomerase IV and DNA gyrazes. But due to the high and incorrect use of these antibiotics, resistance to them has emerged [7]. In recent years, high levels of *qnr*-mediated resistance among fluoroquinolones and quinolones-resistant Enterobacteriaceae isolates have been reported, making the treatment of these strains more complicated [8].

Molecular typing is an important infection control tool to evaluate relationships between different isolates of bacteria. It helps us determine the sources of contamination, investigate the distribution status of pathogens, know how many pathogens changed over time, choose the best treatment of diseases, and reduce the risks of antibiotic resistance [9]. The earlier traditional methods were based on phenotypic typing techniques while modern molecular approaches, particularly DNA-based methods, have become potentially powerful methods in microbial typing. These include analysis of plasmid profiles, ribotyping, RFLP (restriction fragment length polymorphism), AFLP (amplified fragment length polymorphism), MLST (multilocus sequence typing), RAPD (random amplification of polymorphic DNA), MLVA (multiplelocus variable number tandem repeat analysis), VNTR (variable number tandem repeat), rep-PCR (repetitive extragenic palindromic), ERIC-PCR (enterobacterial repetitive intergenic consensus PCR), microarray, and PFGE (pulse-field gel electrophoresis). Among these, PFGE as a gold standard method is most commonly used. This technique has the high power of typing, repeatability, and differentiation [10]. The aims of this work were the molecular characterization of antibiotic resistance and genotyping of *K. pneumoniae* strains isolated from clinical specimens in a major referral hospital in Tehran, Iran.

## 2. Methods

### 2.1. Samples and Bacterial Isolation

During one year, a total of 100 clinical human samples including urine, blood, and sputum were collected from a major referral hospital in Tehran (capital of Iran), Iran. Midstream urine was collected in sterile condition to decrease potential bacterial, cellular, and artifactual contamination. The bacterial isolates were identified as *K. pneumoniae* by standard microbiological and biochemical methods [2]. In addition, all of the isolates were confirmed using the polymerase chain reaction (PCR)-based amplification of the *16SrRNA* gene [11, 12].

### 2.2. Antibiotic Susceptibility Pattern

Antibiotic susceptibility testing of *K. pneumoniae* isolates was performed according to the Kirby–Bauer disk diffusion method. The Mueller–Hinton agar (Merck, Germany) medium was used for this purpose. Principles of the Clinical and Laboratory Standards Institute (CLSI) guidelines were used for this purpose to assess antibiotic resistance using trimethoprim (5 *μ*g), cefoxitin (30 *μ*g), ciprofloxacin (5 *μ*g), nalidixic acid (30 *μ*g), nitrofurantoin (300 *μ*g), tetracycline (30 *μ*g), streptomycin (10 *μ*g), gentamicin (10 *μ*g) and meropenem (10 *μ*g) [13]. All of the inoculated plates were aerobically incubated at 37°C for 18–24 h in an aerobic atmosphere. *K. pneumoniae* ATCC 4352 was used as a quality control organism [13].

### 2.3. Molecular Characterization of Antibiotic Resistance

The DNAs of *K. pneumoniae* were extracted using a DNA extraction kit (Bioneer, Daejeon, South Korea) according to the manufacturer's instructions. PCR was used to amplify the *gyrA* gene in nalidixic acid and ciprofloxacin-resistant strains as described previously [14]. Sequencing was done for the detection of probable mutation in the *gyrA* gene among the isolates.

PMQR determinants (*qnrA*, *qnrB*, and *qnrS*) were amplified using the primer sets listed in Table 1. Amplification was performed using a thermal cycler (Eppendorf, Hamburg, Germany) for 30 cycles. The PCR amplification reaction mixture consisted of 1 *μ*l of each primer, 2.5 *μ*l PCR buffer (10x), 0.7 *μ*l MgCl_2_ (50 mM), 0.7 *μ*l of each 10 mM dNTP (MBI Fermentas, Vilnius, Lithuania), 0.5 *μ*l of 5 U Pfu DNA polymerase, and 1 *μ*l of sample DNA. PCR assays consisted of initial denaturation at 94°C for 5 min, denaturation at 94°C for 1 min, annealing of primers at 51–53°C for 1 min, primer extension at 72°C for 1 min, and the final extension at 72°C for 7 min (Table 1) [15]. To ascertain the expected sizes of the amplicons, the PCR products were separated by electrophoresis at 100 V for 2 h on 1.5% (*w*/*v*) agarose gels and visualized using an ultraviolet (UV) transilluminator (Tanon, Shanghai, China).

### 2.4. Pulsed Field Gel Electrophoresis

The clonal relatedness of *K. pneumoniae* isolates was determined by the PFGE method. In this regard, XbaI restriction endonuclease (Fermentas) was used to digest genomic DNAs. All the PFGE steps were accomplished following the CDC-standardized procedure used by all PulseNet laboratories with some changes. The genetic relatedness of *K. pneumoniae* isolates was evaluated by PFGE analysis as previously described [16].

### 2.5. Statistical Analysis

Statistics were subjected to Microsoft Office Excel (version 15; Microsoft Corp., Redmond, WA, USA). Statistical analysis was performed by means of the SPSS 21.0 statistical software (SPSS Inc., Chicago, IL, USA). The chi-squared test and Fisher's exact two-tailed test were applied to measure any significant relationship. A *P* value <0.05 was considered a statistically significant level.

## 3. Results

### 3.1. Bacterial Isolates and Antibiotic Resistance

A total of 100 hospital-acquired infections harbored *K. pneumoniae* and were subjected to the study. All positive strains were also approved using the *16SrRNA*-based PCR amplification. Urine samples (56%) had the highest prevalence of *K. pneumoniae*, while blood (12%) had the lowest. A statistically significant difference was seen between the types of samples and the prevalence of *K. pneumoniae* (*P* < 0.05).

Table 2 characterizes the antibiotic resistance pattern of *K. pneumoniae* strains isolated from different types of hospital-acquired infections. The antibiotic susceptibility pattern revealed a high level of resistance to trimethoprim (63%) followed by nalidixic acid (53%) and cefoxitin (45.45%), while the low level of resistance was against meropenem (20%), gentamycin (25%), and streptomycin (30%). A statistically significant difference was seen between the types of samples and the prevalence of antibiotic resistance (*P* < 0.05).

### 3.2. Molecular Characterization of Antibiotic Resistance

Molecular analysis detected mutation in the *gyrA* gene in 51% of quinolone-resistant *K. pneumoniae*. The sequencing showed that the mutant isolates carried point mutations in the *gyrA* quinolone resistance determining regions (QRDR) at codon 83 or 87 which leads to the substitution of different amino acids in *gyrA* protein.

The results of DNA amplification by the PCR method based on the primers described in Table 3 showed the presence of 516-bp, 526-bp, and 417-bp fragments for the PMQR genes specifically amplified by *qnrA*, *qnrB*, and *qnrS* primers, respectively. According to the PCR results, 7%, 18%, and 38% of isolates contained *qnrA*, *qnrB*, and *qnrS*, primers respectively. A statistically significant difference was seen between types of samples and the prevalence of PMQR genes (*P* < 0.05). Moreover, 10% (10/100) of the isolates were found to be positive for both genes.

According to the susceptibility report, among nalidixic acid resistant strains 60.5%, 50%, and 42.9% of isolates contained *qnrA*, *qnrB,* and *qnrS*, respectively. While, 57.1%, 50%, and 39.5% harbored *qnrA*, *qnrB,* and *qnrS*, respectively. Moreover, among ciprofloxacin-resistant strains, *qnrA* was the most frequent PMQR gene, while, *qnrS* and *qnrB* were the most frequent PMQR genes among nalidixic acid susceptible strains. The *qnrS* and *qnrA* genes were found to be relatively higher among nalidixic acid and ciprofloxacin resistance isolates (Table 3).

### 3.3. Pulsed Field Gel Electrophoresis

**Figure 1:**
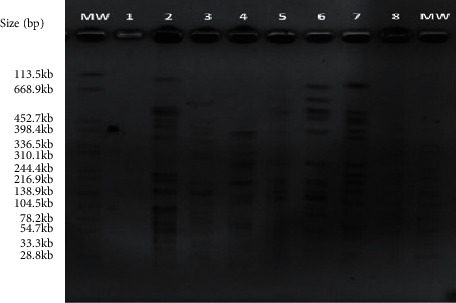
PFGE patterns of some representative clinical samples of *K. pneumoniae*; M: molecular weight marker (*Salmonella braenderup*); line 1: molecular weight marker (*Salmonella braenderup*); line 3: strain no 46 (palsotype D); line 4: strain no 32 (palsotype E); line 5: strain no 7 (palsotype H); line 6: strain no 38 (palsotype O); line 7: strain no 15 (palsotype B); line 8: strain no 14 (palsotype A); line 9: strain no 41 (palsotype A); line 10: molecular weight marker (*Salmonella braenderup*).

**Figure 2:**
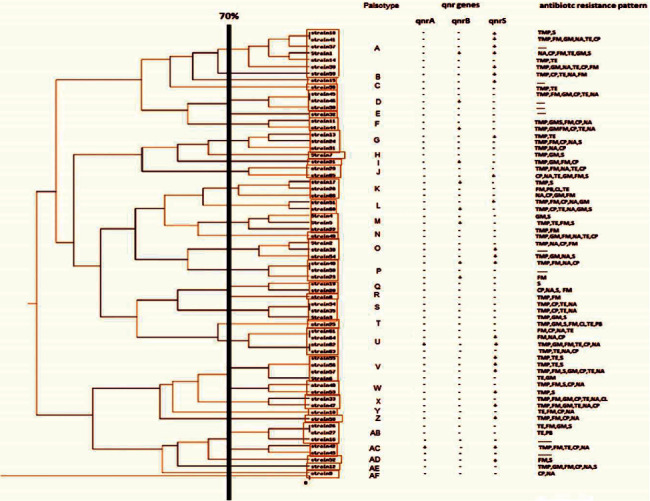
Dendrogram of clinical samples of *K. pneumoniae*.

From 100 *K. pneumoniae* strains, 66 isolates gave an acceptable band pattern and were subjected to PFGE. PFGE results showed that *K. pneumoniae* strains had 31 different genetic patterns that were arbitrarily named A-AF (Figures 1 and 2). The highest number [7] was in category *A*, and the lowest number [1] in categories B, C, E, H, I, N, R, T, Y, Z, AD, AE, and AF. Most of the strains (75%) were isolated from urine, which was mostly assigned the A-palsotype. The highest antibiotic resistance rates were also observed among the isolates recovered from urine specimens. More than 43.9% of the strains had *qnr* genes, with the highest number of them clustered in palsotype A (Figure 2). A statistically significant difference was seen between types of samples and the prevalence of PMQR genes (*P* < 0.05).

## 4. Discussion

*K. pneumoniae* is one of the most important opportunistic pathogenic bacteria in people with immunodeficiency and underlying diseases [17]. The bacterium has been recognized as one of the most common causes of acquired infections in the hospital, with multiple drug resistance. The emergence and development of drug resistance is a major problem in hospitals, and infections caused by drug-resistant bacteria cause a high mortality rate [18]. Pathogenic bacteria that cause nosocomial infections are resistant to many antibiotics, so it is important to recognize and control the spread of multidrug-resistant microbial infections [19]. In recent years, plasmid-associated resistance to quinolones is frequently reported among Enterobacteriaceae isolates in several studies around the world, although the number of reports of the prevalence of *qnr* genes in this family in Iran has been limited to a few studies [20].

In this study, the most antibiotic resistance in *K. pneumoniae* isolates was related to trimethoprim (63%) followed by nalidixic acid (53%) and cefoxitin (45.45%) antibiotics. The lowest rate of resistance was seen toward meropenem (20%), gentamycin (25%), and streptomycin (30%). Recently in Iran, Dehghan et al. in 2016 examined the pattern of antibiotic resistance in 120 strains of *K. pneumoniae* isolated from urine specimens [21]. The antibiotic resistance rates were 23.8% to ciprofloxacin, 24.6% to nalidixic acid, 11.5% to cefoxitin, 29.2% to gentamicin, and 35.4% to tetracycline. Their results for gentamicin and tetracycline are consistent with the present study, but the rates of resistance against ciprofloxacin and nalidixic acid have increased in our study [21].

Also, in another study, Latifpour et al. in 2016 examined the prevalence and antibiotic resistance pattern of 150 strains of broad-spectrum beta-lactamases producing *K. pneumoniae* isolated from UTI in hospital and outpatients. Resistance patterns among their isolates were trimethoprim (61%), gentamycin (59%), nitrofurantoin (55%), nalidixic acid (72%), and ciprofloxacin (60%). With an exception of gentamicin and nitrofurantoin, their results are consistent with our findings [22].

Ranjbaran et al. in 2013 evaluated the antibiotic susceptibility pattern of 50 isolates of *K. pneumoniae* for samples of UTI in a number of patients in intensive care. In this study, the antibiotic resistance of ciprofloxacin was 42.4%, nitrofurantoin was 38%, and gentamicin was 21.2% indicating the results of this study were not consistent with our finding [23]. The differences in the results may be due to the level of health in the area studied, the difference in the sampling method, the difference in the geographical area, diet, the use of infection control tools in different parts of hospitals, or because of the high consumption of some antibiotics in these patients.

The sequencing of the *gyrA* gene showed the mutations in codons 83 and 87, resulting in the substitution of different amino acids in *gyrA* protein among the quinolone-resistant resistant *K. pneumoniae*. Previously, several studies in our country have shown this mutation can cause the replacement of the amino acid serine to phenylalanine (Ser83 ⟶ Phe), serine to tyrosine (Ser83 ⟶ Tyr), and serine to leucine (Ser83 ⟶ Leu) [24, 25].

Kareem et al. reported that 40% of quinolone-resistant *K. pneumoniae* strains had the mutation of the *gyrA* gene [24]. In another study conducted by Norouzi et al. in Iran, the substitution of Ser 83 ⟶ Ile and Ser 83 ⟶ Phe in the *gyrA* gene was reported among *K. pneumoniae* strains [20]. In Iraq, as a neighboring country, the substitution of Ser83 to Leu has been reported as the most common mutation in *gyrA* among all quinolone-resistant *K. pneumoniae* isolates, followed by Asp87 ⟶ Asn [24]. Huang et al. detected point mutations in *gyrA* among 95% of ciprofloxacin-resistant *Klebsiella* strains isolated from China. Huang et al. in a recent study showed in [26].

The frequency of *qnr* plasmid-mediated genes showed that among 100 isolates of *K. pneumonia*e 7%, 18%, and 38% of isolates contained *qnrA*, *qnrB,* and *qnrS*, respectively. A statistically significant difference was seen between types of samples and the prevalence of PMQR genes (*P* < 0.05). Moreover, 10% (10/100) of the isolates were found to be positive for both genes.

Salimizand et al., in 2021, studied the frequency of *qnrA*, *qnrB*, and *qnrS* genes among *K. pneumoniae* isolates obtained from hospitalized patients. The results obtained in that study showed that 52%, 22 25%, and 23% of isolates of *K. pneumoniae* contained the *qnrB*, *qnrA*, and *qnrS* genes, respectively [27].

Taraghian et al. studied plasmid-mediated quinolone resistance in quinolone-resistant *K. pneumoniae* isolates in Iran. The results of their study showed that the most common qnr gene was *qnrB* (84.7%), followed by *qnrS* (65.2%) and *qnrA* (22.2%). The frequencies of *qnr* found in their study were higher than those obtained from our study [28].

Perez-Lopez et al., in 2020, examined plasmid-mediated quinolone resistance determinants in *K*. *pneumoniae* strains in Qatar. The rate of *qnr* A/B/E/S (plasmid gene was 45.3% in *K. pneumoniae* isolates) indicated the higher frequency of *qnr* plasmid genes compared to our study [29].

Therefore, findings suggest that the emergence of PMQR helps to rapidly increase the spread of bacterial resistance to fluoroquinolones, which requires continuous monitoring of antibiotic use [30].

PFGE is one of the genotypic methods used for bacterial typing and is superior to other molecular methods due to its high differentiation in different bacterial strains. This method is able to differentiate the large genome of organisms [31]. Nowadays, using different methods of typing in hospitals, the prevalence of many hospital infections is prevented, and for this reason, the economy and health of various communities have contributed greatly [5, 32].

In this study, PFGE results showed 31 different genetic patterns among the strains that were arbitrarily named as A-AF. The highest number [7] was seen in category A and the lowest number [1] in categories B, C, E, H, I, N, R, T, Y, Z, AD, AE, and AF. Pons et al. classified ESBL among 19 isolates of *K. pneumoniae* recovered from the patients with bacteremia and UTI in Mozambique [33]. PFGE analysis showed that all isolates belonged to different clusters. Moreover, Hashemi et al. examined the genetic pattern and determination of the level of *oqxA* gene expression in 111 clinical isolates resistant to antibiotics of *K. pneumoniae* isolated from inpatients. They used the PFGE for genotyping resistant strains. Fifteen patterns of PFGE including 3 main clusters A, B, and C were identified [34].

Ashayeri–Panah et al. investigated the genetic profile of 54 clinical isolates of *K. pneumoniae* for subtyping using PFGE and RAPD methods. The results of that study showed 30 bands in the range of 15–7 bp and at the similarity level of 70%, 22 large clusters (W-A) at the similarity level of 85%, and 42 different groups were shown [35]. Han et al. used 10 strains of *K. pneumoniae* to optimize subtyping using the PFGE method. In this method, the XbaI and AvrII enzymes were used. Both of these enzymes had a *D*-value greater than 99% for *K. pneumoniae* strains. The results of this study showed high repeatability and typography for both enzymes and indicated that electrophoresis parameters for each enzyme were proposed based on the size of limiting enzyme segments for the used equipment in order to obtain optimal results. The results of some studies are consistent with the results obtained in our study in some patterns [36].

Drakhshan et al. examined the genomic typing using PFGE and MLVA methods and examined the genetic diversity of the *bla*_CTX-M-1_ gene in 200 clinical isolates of *K. pneumoniae*. The PFGE and MLST differentiated the strains into 64 and 44 genotypes, respectively, indicating a high degree of differentiation of PFGE compared to the MLVA method [37]. Also, Cubero et al. described the clonal development of 98 clinical isolates of *bla*_oxa-1_ beta-lactamase-producing *K. pneumoniae* in a hospital in Spain. The relationship between *K. pneumoniae* strains was determined by PFGE and MLST. A distinct pattern of PFGE was observed with 5 different subtypes, and all isolates had a common pattern in the PFGE, which was characterized by the sequence of type 14 [38].

In Salimizand et al.'s study that was carried out on 35 MDR *K. pneumoniae* isolates recovered from outpatient and adolescent patients, PFGE analysis identified 18 clusters with clonal dependence in some cases [27]. Furthermore, Chung et al. used PFGE and MLST for the genetic classification of 73 isolates of *K. pneumoniae* from serotype k1, which induces invasive abscess in the liver. More than 94% of the isolates were clustered in PFGE in a large group with a similarity of ≥70%. Within this group, 8 subgroups with ≥80% similarity were clustered. The findings of that study showed a very similar pattern in the PFGE method too which our study is in contrast with [39].

In a very recent study in Taiwan, over a period of 8 months, the molecular epidemiology of 31 isolates of ESBL-producing *K. pneumoniae* was investigated, in which PFGE differentiated the strains into 16 distinct genotypes and 5 clusters. In 20 isolates, the coefficient of similarity was more than 80%, and in 11 isolates, between each cluster, a high level of genetic heterogeneity was observed, which is consistent with our study [40].

## 5. Conclusion

The results of this and similar studies indicated that the frequency of resistance to various antibiotics including quinolones in *K. pneumoniae* isolates is increasing. The presence of *qnr* (*S* and *A*) genes and point mutation of the *gyrA* gene were likely to be responsible for the resistance toward nalidixic acid and ciprofloxacin in our strains. Also, genotyping using PFGE indicated that the *K. pneumoniae* strains isolated in this study belonged to diverse clones.

## Figures and Tables

**Table 1:** PCR primers were used in the study for the detection of *qnr* genes in *K. pneumoniae* isolates.

Primer/target amplicon	Primer sequence (5′ ⟶ 3′)	Amplicon size (bp)	Annealing temperature (°C)
*qnrA*	F: ATT TCT CAC GCC AGG ATT TG R: GAT CGG CAA AGG TTA GGT CA	516	53

*qnrB*	F: GTT GGC GAA AAA ATT GAC AGA A R: ACT CCG AAT TGG TCA GAT CG	526	53

*qnrS*	F: ACG ACA TTC GTC AAC TGC AA R: TTA ATT GGC ACC CTG TAG GC	417	51

**Table 2:** Characteristics of clinical samples based on the antimicrobial resistance pattern and molecular typing.

Type of antibiotic	Antibiotic resistance pattern (%)			
	Total (*n* = 100)	Urine (*n* = 56)	Sputum (*n* = 32)	Blood (*n* = 12)
Meropenem (10 *μ*g)	20 (20)	17 (30)	1 (3.1)	2 (16.7)
Trimethoprim (5 *μ*g)	63 (63)	56 (100)	6 (18.8)	1 (8.3)
Cefoxitin (30 *μ*g)	50 (50)	46 (82.1)	3 (9.4)	1 (8.3)
Ciprofloxacin (5 *μ*g)	49 (47)	42 (75)	5 (15.6)	2 (16.7)
Nalidixic acid (30 *μ*g)	53 (53)	46 (82.1)	5 (15.6)	2 (16.7)
Nitrofurantoin (300 *μ*g)	42 (42)	38 (67.9)	4 (12.5)	0 ()
Tetracycline (30 *μ*g)	40 (40)	36 (64.3)	3 (9.4)	1 (8.3)
Streptomycin (10 *μ*g)	30 (30)	27 (48.2)	3 (9.4)	0 (0)
Gentamycin (10 *μ*g)	25 (25)	23 (41.1)	2 (6.2)	0 (0)

**Table 3:** Distribution of *qnr*-genes among quinolone resistance.

Antibiotic	Pattern no (%) 100	*qnrS*-positive no (%) 38	*qnrB*-positive no (%) 18	*qnrA*-positive no (%) 7	*qnrB* and *qnrS*-positive no (%)	*qnrA* and *qnrS*-positive no (%)
Nalidixic acid	R (*n* = 53)	23 (60.5)	9 (50)	3 (42.9)	3 ()	3 ()
	S (*n* = 47)	15 (39.5)	9 (50)	4 (57.1)	2 ()	2 ()

Ciprofloxacin	R (*n* = 49)	19 (50)	9 (50)	4 (57.1)	3 ()	3 ()
	S (*n* = 51)	19 (50)	9 (50)	3 (42.9)	2 ()	2 ()

## Data Availability

The datasets used and/or analyzed during the current study are available from the corresponding author upon reasonable request.
